# Tumor‐Infiltrating Lymphocytes in Breast and Female Genital Tract Cancers: Overlooked Potential and Unexplored Frontiers

**DOI:** 10.1002/cam4.71023

**Published:** 2025-07-07

**Authors:** Kristijan Skok, Umberto Maccio, Spencer D. Martin, Konstantin Bräutigam

**Affiliations:** ^1^ Diagnostic and Research Institute of Pathology, Medical University of Graz Graz Austria; ^2^ Institute of Biomedical Sciences, Faculty of Medicine University of Maribor Maribor Slovenia; ^3^ Department of Pathology and Molecular Pathology University Hospital of Zurich Zurich Switzerland; ^4^ Department of Pathology and Laboratory Medicine University of British Columbia Vancouver British Columbia Canada; ^5^ Centre for Evolution and Cancer Institute of Cancer Research London UK

**Keywords:** breast cancer, endometrial cancer, fallopian tube, immunotherapy, ovarian cancer, review, tumor microenvironment (TME), tumor‐infiltrating lymphocytes (TILs), vulvar cancer

## Abstract

**Background:**

The growing success of cancer immunotherapies has led to significant advances in oncology. However, despite these promising developments, cancer‐related mortality remains high for common cancer types such as breast and lower female genital tract cancers.

**Method:**

Here, we synthesize recent findings on the prognostic relevance of tumor‐infiltrating lymphocytes (TILs) in breast, endometrial, tubo‐ovarian, and vulvar cancer. Our analysis covers the relationship between TIL counts and density, immune cell subtype combinations, immunotherapy approaches, and patient outcomes.

**Results:**

High TIL infiltration, especially CD8^+^ T‐cells, generally correlates with improved outcomes such as in endometrial cancer (especially the *POLE*‐ultramutated subgroup), invasive breast cancer, and ovarian epithelial tumors. However, in ductal carcinoma in situ (DCIS) of the breast, elevated TIL counts are linked to a worse prognosis. Ethnicity, the tumor microenvironment (TME), and molecular profiles further complicate the prognostic utility of TILs.

**Conclusions:**

TIL‐based therapies have shown potential in personalized immunotherapy, particularly in recurrent, refractory ovarian cancer. Limited research on rarer gynecologic tumors hinders broader clinical applications.

## 1 Introduction

Tumor‐infiltrating lymphocytes (TILs) have become a hot topic in daily clinical routine, be it the routine of a pathologist, gynecologist, or oncologist [1]. While the role of TILs in breast cancer (BC) has been investigated for years [2], their significance in cervical, endometrial, tubo‐ovarian, and vulvo‐vaginal cancers is less explored.

### 1.1 TILs and the Tumor Microenvironment

**FIGURE 1:**
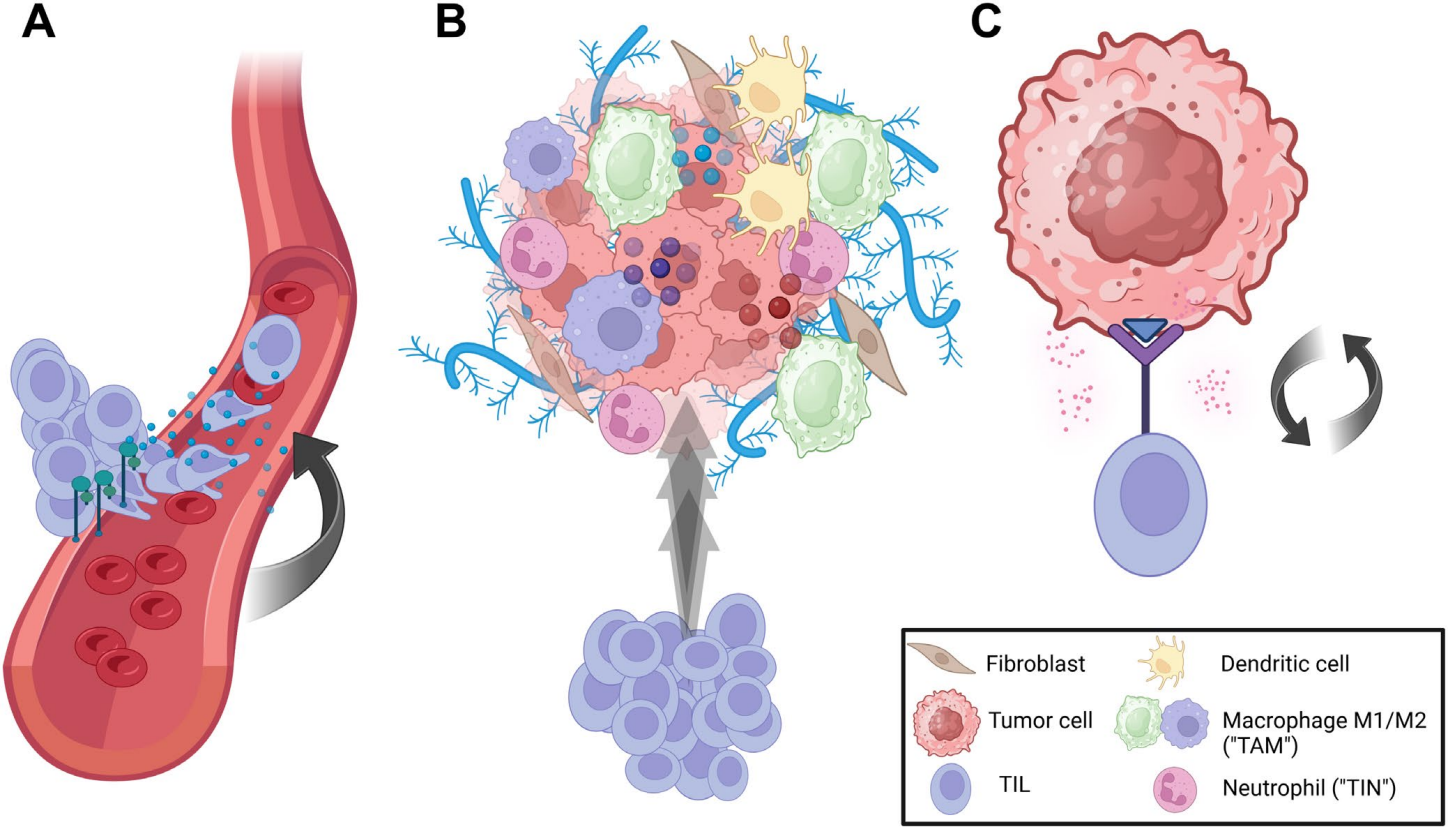
Multi‐step mechanism of tumor‐infiltrating lymphocytes (TILs). (A) *Migration*: TILs migrate via the bloodstream to reach tumor sites, guided by cytokines (blue dots) and adhesion molecules (green). (B) *Tumor recognition*: On site, TILs invade the collagen matrix (light blue) of the tumor microenvironment (TME) and are confronted with a more or less hostile local cell infiltrate, e.g., cells of the innate immunity such as (tumor‐associated) macrophages (TAMs), tumor‐infiltrating neutrophils (TINs), and dendritic cells. TAMs can be polarized into an M1‐ (green) and M2‐subgroup (purple). While M1‐macrophages secrete pro‐inflammatory cytokines (red and purple dots), e.g., Tumor necrosis factor (TNF)‐α, M2‐macrophages mediate anti‐inflammatory action (light blue dots). (C) *Effect*: TILs recognize and interact with tumor‐associated antigens (blue triangle), leading to a release of cytotoxic granules (pink dots). Created in BioRender.com.

The tumor microenvironment (TME) is a dynamic system where tumor cells interact with stromal cells [3], blood vessels, the extracellular matrix, and immune cells [4]. Within the TME, various immune cells can either suppress tumor growth or promote tumor growth by suppressing the anti‐tumor response [5]. Key cellular players include tumor‐associated neutrophils (TANs) [6], tumor‐associated macrophages (TAMs) [7], and TILs [8], all of which may interact with tumor cells and with each other through a cascade of complex mechanisms (Figure 1). For example, TILs, including B‐, T‐, and Natural Killer (NK)‐cells, infiltrate tumor stroma (known as stromal TILs, “sTILS”) and tumor nests (intraepithelial TILs, “ieTILS”) [9]. Intratumoral T‐cells may directly recognize and kill tumor cells via binding to an immunogenic peptide within the major histocompatibility complex (MHC). Moreover, NK cells can recognize and kill tumor cells that attempt to evade the T‐cell response by downregulating MHC molecules [10]. B‐cells can act through various mechanisms to either inhibit or promote tumor growth [11]. Multiple studies have demonstrated that TILs play important roles in the process of carcinogenesis and have therapeutic and prognostic implications [12]. However, tumors deploy mechanisms to evade immune attack, causing TILs to become dysfunctional [13]. This immune escape involves metabolic shifts, hypoxia, and immune checkpoint (IC) overexpression, which allow tumors to remain undetected by the immune system [14].

### 1.2 Immunotherapy and TILs

Multiple strategies have been employed to attempt to activate and enhance the anti‐tumor immune response. ICs such as cytotoxic T‐lymphocyte associated protein 4 (CTLA‐4) and programmed death‐1 (PD‐1) expressed on T‐cells, and its ligand programmed death‐ligand 1 (PD‐L1) expressed on tumor and macrophages are critical for evading anti‐tumor immunity, with PD‐L1 expression often linked to immune evasion in tumors [15]. Restoring TIL function through IC inhibitors (ICIs) has fundamentally altered cancer therapy, and PD‐L1 expression is a standard biomarker to predict ICI response in many tumors, including lung carcinoma, gastroesophageal adenocarcinoma, urothelial carcinoma, squamous cell carcinoma (SCC) of the cervix, and triple‐negative breast cancer (TNBC) [16, 17]. Importantly, ICIs are approved for therapy in PD‐L1^+^ TNBC [18], where improved survival has been demonstrated in patients with higher numbers of TILs before treatment [19]. In BC, the tumor's molecular subtype significantly affects its interaction with the immune system. For example, TNBC and HER2^+^ BC tend to have higher numbers of TILs than hormone receptor (HR)‐positive tumors, indicating a more robust immune response in these subtypes [20, 21]. Apart from ICI, TIL therapy has become a valid treatment option in cancer care. TIL therapy is a type of cell‐based immunotherapy whereby the patient's own immune cells from the microenvironment of the solid tumors are expanded ex vivo and inoculated back into the patient, often with high‐dose Interleukin (Il)‐2, to kill tumor cells [22].

### 1.3 Significance of TILs in Cancer

Multiple studies (Table S1) have demonstrated that the presence and activity of TILs may influence patient prognosis across various cancer types [23, 24, 25], positioning them as a promising biomarker for predicting immunotherapy response and patient survival [26]. Gaining a thorough understanding of the cellular landscape and function of TILs is essential for advancing cancer immunotherapy strategies and developing personalized treatment approaches [8]. While the key role of TILs in the immune response in BC is well known, the study of TILs in lower female genital tract cancers is still developing. In the following, we synthesize current knowledge on TILs in breast and female genital tract cancers.

## 2 TILs in Breast Cancer

### 2.1 The Prognostic and Predictive Impact of TILs and Their Microenvironment in BC

**FIGURE 2:**
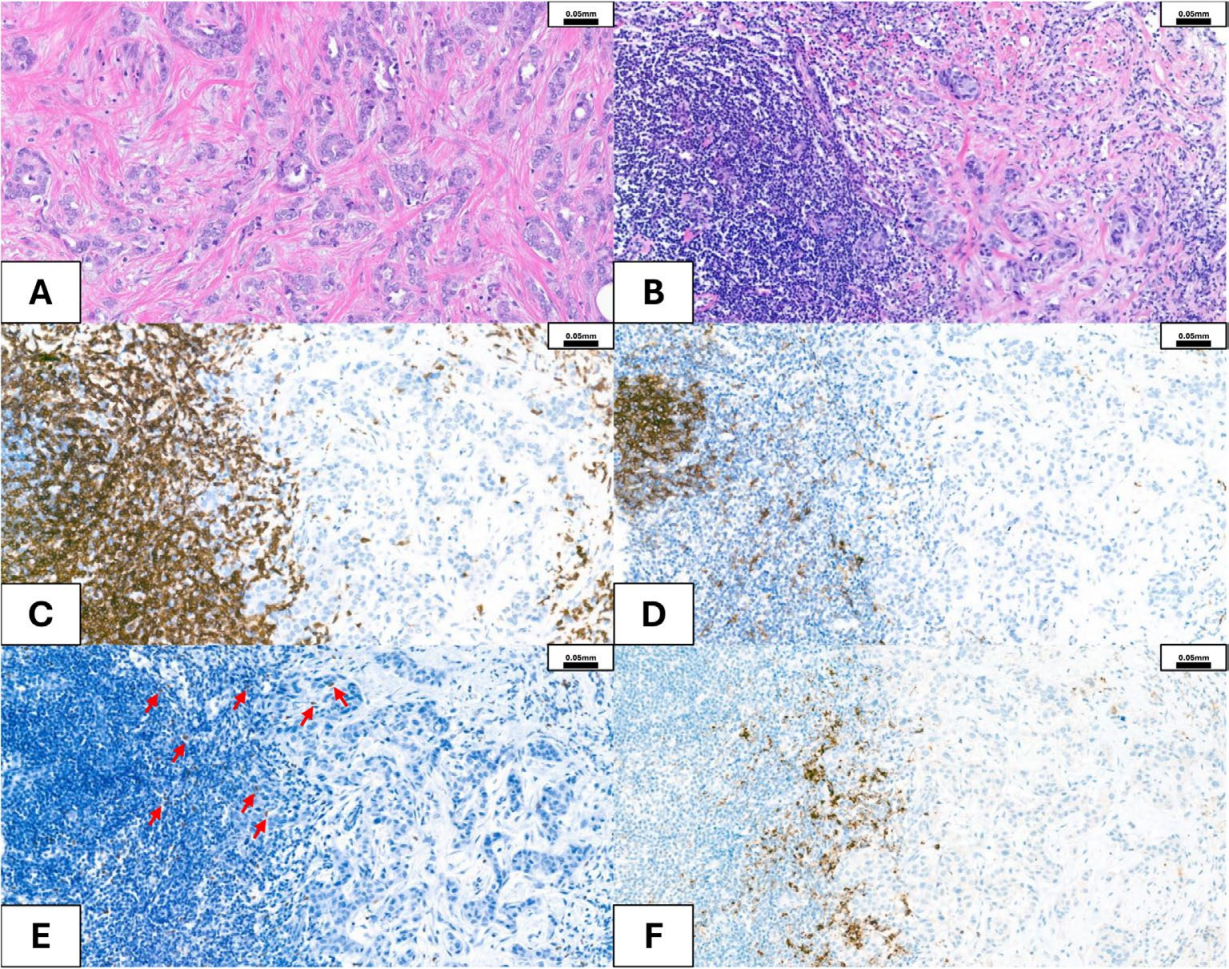
Breast cancer. (A) Moderately differentiated invasive breast cancer (non‐special type, BRE* (Bloom‐Richardson‐Elston)‐Score 3/2/1, Grade 2), hormone receptor (HR) positive (HR+) with no amplification of *HER2* gene, showing very few tumor infiltrating lymphocytes (TILs). (B) Poorly differentiated invasive breast cancer (non‐special type, BRE‐Score 3/3/3, Grade 3), hormone receptor (HR) positive (HR+) with amplification of *HER2* gen, showing numerous peri‐ and intratumoral TILs. (C) CD3^+^ stain showing numerous T‐lymphocytes. (D) CD20^+^ showing some B‐lymphocytes. (E) FOXP3 showing sparse CD4^+^ T‐regulatory cell (T_reg_) infiltration—red arrows. (F) PD‐L1 immunohistochemical stain (*VENTANA* PD‐L1 (SP142)) showing focal membranous positivity in tumor and in immune cells. *The Bloom‐Richardson‐Elston (BRE)‐score quantifies the amount of gland formation/nuclear features/mitotic activity for breast cancer grading.

In 2010, Denkert et al. identified TILs as a novel independent predictor of pathologic complete response (pCR) to anthracycline/taxane neoadjuvant chemotherapy (NAC) in TNBC, revealing the percentage of TILs as a significant parameter [25]. In 2014, the *International TILs Working Group* published a standardized methodology for TIL evaluation (Table 1), recommending its integration into routine histopathological practice [2]. It has been reported that approximately 40%–50% of TNBC patients achieve a pCR after NAC. Predictive biomarkers like high Ki‐67, PD‐L1 expression, and abundant CD8^+^ TILs are linked to pCR. A prediction model combining these markers may better stratify TNBC patients for NAC response [27]. In early‐stage TNBC, without adjuvant or NAC, cancers with a higher abundance of TIL levels were associated with significantly better survival. Consequently, TIL abundance is a prognostic factor for patients with early‐stage TNBC [28]. Chemotherapy‐naive, young patients with node‐negative (N0) TNBC with high sTILs (≥ 75%) have an excellent long‐term prognosis [29]. In detail, a TIL profile with low CD3^+^, CD4^+^, CD20^+^, and CD56^+^ expression predicts poor response to NAC in TNBC [30]. Ethnic diversity plays a role as well [31], with Asians having a greater amount of TILs in comparison with Caucasians [32]. Moreover, higher levels of TILs correlate with better response to NAC and improved prognosis in both TNBC and HER2^+^ BC (Figure 2A), but not for patients with HR^+^ BC [33]. These patients may potentially benefit from therapy with ICIs (Table 1).

**TABLE 1:** Prognostic and therapeutic relevance of TILs in breast and female genital tract cancers.

Cancer type	Prognostic relevance of TILs	Immunotherapy	Histological TIL assessment
Breast HR^+^ Her2^−^ (luminal)	HR^+^ Her2^−^ BC is less immunogenic than other subtypes; contrasting evidences on prognostic and predictive value of TILs	Not recommended but clinical trials are ongoing	Not routinely recommended)
Breast Her2^+^	Higher TILs correlate with better prognosis and response to NAC	Not recommended	Yes (TIL scoring guidelines available)
Breast TNBC	Higher TILs correlate with better prognosis and response to NAC	Pembrolizumab (PD‐1 inhibitor) + NAC (KEYNOTE‐522) associate with pathological complete response	Yes (TIL scoring guidelines available)
Breast DCIS	Higher TILs may correlate with poor outcomes and ipsilateral invasive carcinoma	Not used for carcinoma in situ	Not routinely required
Endometrial POLE‐mutated	Within the POLEmut subtype, outcomes are so favorable, regardless of immune infiltration, that TIL density is unlikely to impact survival	POLEmut is now also considered to be low risk and unlikely to benefit from adjuvant treatment	No
Endometrial MMRd	Increased CD8^+^ TILs have been found to associate with response to therapy in some studies	MMRd endometrial carcinoma responds very well to ICI. MSI‐H – Dostarlimab ± Chemotherapy (PD‐1 testing), Durvalumab + Chemotherapy (PD‐L1 testing); Pembrolizumab (PD‐1 testing)	No
Endometrial p53abn	Reportedly, increased TIL is associated with improved prognosis in p53abn EC	p53abn EC respond to immunotherapy, even though the mutation burden is an order of magnitude less than POLE‐mutated and MMRd subtypes	No
Endometrial NSMP	TILs in the invasive margin of this subtype have been found to be prognostic of overall survival	NSMP failed to respond to ICI	No
Ovarian HGSC	Higher levels of T‐regulatory cells (T‐regs) in HGSC, both in intratumoral and stromal areas, are associated with significantly increased OS. Some studies support the association of higher T‐reg cell infiltration with improved outcome, while others claim the opposite	Autologous TIL transfer and ongoing trials for refractory, recurrent ovarian cancer	No
Ovarian OCCC	CD8^+^ T‐cell infiltration associated with improved survival and inversely correlated with hypoxia. High TILs (≥ 50/mm^2^) and PD‐L1 positivity (Combined Positive Score) predict shorter disease‐specific survival		
Ovarian endometrioid	Presence of intraepithelial CD8^+^ T‐cells was described to be lower than in serous carcinoma and not associated with improved disease‐specific survival		
Ovarian mucinous	Mucinous ovarian cancer seems to be rather “immune‐cold” but can still demonstrate PD‐L1 protein expression		
Cervical adenocarcinoma	Yes, higher TIL counts are often linked to better prognosis, but the number of TILs was in one study significantly lower in cervical adenocarcinoma in comparison with cervical SCC	Experimental therapies, including TIL‐based approaches, are under investigation	No
Cervical SCC	According to studies, immune infiltration was an independent positive prognosticator for DFS in patients with cervical SCC. Patients with the strongest TIL infiltration showed better DFS		
Vulvar HPV‐dependent SCC	Spatial transcriptomics studies show distinct immune cell infiltration patterns, with upregulated IL‐17 signaling and differences in T cell subsets compared to HPV‐independent vulvar SCC	ICI, PD‐L1 inhibition shows promise in smaller patient cohorts	No
Vulvar HPV‐independent SCC	Significantly more immune cells in HPV‐independent SCC than in HPV‐dependent SCC		
Vaginal	Unclear	Unclear	No

Abbreviations: DCIS, ductal carcinoma in situ; DFS, disease‐free survival; EC, endometrial carcinoma; HGSC, high‐grade serous carcinoma; HR, hormone receptor; ICI, immune checkpoint inhibition; MMRd, mismatch‐repair deficient; NAC, neoadjuvant chemotherapy; NSMP, no specific molecular profile; OCCC, ovarian clear cell carcinoma; OS, overall survival; p53abn, p53 abnormal; PD‐L1, programmed cell death ligand 1; POLE, DNA‐polymerase epsilon; SCC, squamous cell carcinoma; TIL, tumor‐infiltrating lymphocyte; TNBC, triple‐negative breast cancer.

### 2.2 Immune “Hot” and “Cold” BC

RNA‐sequencing allows for the division of tumors into “hot” and “cold” based on immunological characteristics [34]. Hot tumors are immune‐active, with high infiltration of immune cells like cytotoxic T‐cells and elevated expression of immune‐related genes, making them more responsive to immunotherapy (Figure 2B–D). In contrast, cold tumors have low immune cell infiltration and minimal immune activity, showing reduced expression of immune‐related genes, and are generally less responsive to immunotherapy [34]. Analysis of *The Cancer Genome Atlas* (TCGA) datasets revealed that invasive breast carcinoma of no‐special‐type (NST) and estrogen receptor (ER) positive tumors were significantly associated with cold features. Similarly, computational spatial TIL “mapping” using digital pathology identified invasive lobular carcinomas as cold tumors, while TNBC were hot tumors [35]. It has been postulated that the predictive value of low TILs on nonresponse may facilitate screening patients with HR^+^ HER2^−^ or TNBC who may not benefit from NAC [36]. In another study, patients with HER2^+^ luminal B BC displaying high‐level FOXP3^+^ TILs (regulatory T cells) showed shorter disease‐free survival (DFS). Interestingly, a high CD8^+^ “mTILs” (TILs in the tumor margin)/CD68^+^ TAMs ratio was found to be associated with a strong response to therapy with trastuzumab [37]. The HER2‐low BC subtype (which shares more similarities with HER‐negative tumors) has relatively low TILs [38, 39]. Other studies showed that TILs decreased in metastatic tumors, particularly in patients who relapsed after adjuvant chemotherapy. Changes in TILs from primary to metastatic sites could be a prognostic factor after recurrence [40]. With the rise of NK‐cell or CAR‐NK‐cell therapy [41, 42], NK cells are gaining more and more attention. Although present at relatively low frequencies in the TME, NK cells have been consistently associated with improved clinical outcomes across multiple solid tumor types [43, 44]. In HER2^+^ BC models, NK cells played a key role in mediating the therapeutic efficacy of trastuzumab through antibody‐dependent cellular cytotoxicity [45, 46]. A recent study reported unique neighborhood compositions surrounding NK cells in HER2^−^ versus HER^+^ BC, where NK cells from HER2^−^ tumors were more frequently found proximal to tumor cells, whereas those from HER2^+^ tumors were more frequently proximal to CD3^+^ T‐cells [47].

### 2.3 Single‐Cell Analysis Dissects the BC Immune Microenvironment

Single‐cell analysis is becoming more widely available [48], offers insights often obscured in bulk analyses, and is valuable for dissecting cellular heterogeneity [49, 50]. Single‐cell atlases [51, 52, 53] could reveal the cellular composition of the “normal” breast, an important reference for further studies. While single‐cell RNA sequencing (scRNA‐seq) excels at resolving cellular heterogeneity by capturing gene expression profiles at the level of individual cells, it lacks the spatial information necessary to fully characterize how cells interact and organize within the TME. Spatial transcriptomics (ST) [54, 55] addresses this gap by preserving the in situ localization of RNA transcripts, enabling direct measurement of gene expression within tissue sections and the discovery of cellular neighborhoods [48]. Kumar et al. [52] identified in breast tissue from 126 women 12 major cell types and 58 distinct cell states, organized into four principal spatial tissue domains. The work identified a rich and diverse population of tissue‐resident immune cells distributed throughout lobular and ductal regions. These cells predominantly expressed the residency marker RUNX3 and were largely spatially independent of vascular structures, an important implication for BC immunotherapy. Through detailed analysis of a comprehensive single‐cell transcriptomic atlas of the human breast [56], derived from over 800,000 cells collected from 55 individuals, 41 distinct cellular subclusters were found across epithelial, immune, and stromal compartments. Notably, immune cells from individuals carrying *BRCA1* or *BRCA2* mutations exhibited a unique transcriptional profile consistent with immune exhaustion. These results suggest that mechanisms of immune evasion may be active in histologically normal tissue long before the emergence of malignancy [56].

Single‐cell studies have identified tumor‐specific T‐cell clusters exhibiting gene expression signatures associated with inflammatory signaling, hypoxia, and metabolic stress, likely reflecting adaptive responses to the tumor milieu in BC [49]. Furthermore, in triple‐negative BC, single‐cell transcriptomics has elucidated through predicted ligand–receptor interactions that stromal cell populations, such as CAFs and perivascular‐like cells, may contribute to immune suppression by reprogramming TILs [57]. In an integrative scRNA‐seq study [49], researchers analyzed over 45,000 immune cells from eight breast carcinomas alongside matched samples from normal breast tissue, peripheral blood, and lymph nodes. While immune cell profiles in normal and tumor tissues were broadly similar, the TME exhibited distinct continuous phenotypic expansions as defining characteristics of T‐cells and myeloid cells—the primary cellular targets in cancer immunotherapy. In T‐cells, this phenotypic complexity was largely driven by activation through the T‐cell receptor (TCR) and shaped by TCR‐dependent interactions with the TME [49]. An RNA‐sequencing atlas of the BC TME [53] defined six distinct NK‐cell subsets and demonstrated that NK‐cell heterogeneity parallels epithelial cell diversity. To investigate cancer‐immune interactions, the authors developed *InteractPrint*, a computational framework that links epithelial heterogeneity to immune cell engagement. *T cell InteractPrint* successfully predicted responses to ICI in two neoadjuvant anti‐PD‐1 BC trials, outperforming PD‐L1 as a biomarker (AUC = 0.82 and 0.83 vs. 0.50 and 0.72) [58]. NK cells exhibit anti‐tumor potential by secretion of key cytokines such as IFN‐γ, TNF‐α, and GM‐CSF and by directly binding to tumor cells via their activating receptors to induce apoptosis [59].

### 2.4 TILs in Ductal Carcinoma In Situ (DCIS) and BC Subtypes

In some subtypes of BC and precursors, increased TILs have been associated with worse outcomes. For example, multiple studies of ductal carcinoma in situ (DCIS) have found that the presence of TIL was associated with subsequent ipsilateral invasive carcinoma and/or poor outcomes [60, 61, 62]. One might assume that these results indicate that TILs promote BC invasion. However, it is important to recognize that TIL associations do not necessarily indicate causation of poor outcomes. Indeed, TILs get recruited to a tumor site once the basement membrane is disrupted and microinvasion or frank invasion occurs [60, 63]. Moreover, increased TILs have been associated with features that lead to basement membrane disruption in DCIS, like larger size, higher grade, and comedonecrosis [60, 61, 62]. Thus, it is likely that TILs serve as markers of basement membrane disruption and occult invasion in DCIS (thereby associating with poor outcome), but their function in DCIS is to attack early invasive carcinoma. Similarly, in ER^+^/HER2^−^ BC, increased TILs were found to be significantly associated with a poor response to neoadjuvant endocrine therapy [64]. When the specific subtypes of the TILs were assessed, the authors found that the increase in TILs in poor responders was primarily due to increased regulatory T‐cells without accompanying increases in other T‐cell subtypes. Computational deconvolution of bulk tumor transcriptomes showed that the estimated intratumoral TIL count was associated with increased immune response and (cancer) cell proliferation in ER^+^/HER2^−^ and better survival in HER2^+^ and TNBC subtypes, but not always with pCR after NAC [65].

Together, these findings highlight the nuance of using TIL quantity as predictive or prognostic markers, and they show that careful interpretation is required to avoid confounding factors, avoid assigning causation to correlative studies, and interpret TIL quantity in the context of TIL subtype analysis.

### 2.5 TIL Quantification in BC

In recent years, a significant number of studies centering on artificial intelligence (AI) and digital pathology have been published [66, 67, 68, 69], including efforts to develop quantitative methods for TIL assessment [69, 70, 71]. The *International Immuno‐Oncology Biomarker Working Group on Breast Cancer* has thoroughly examined machine learning and image analysis, addressing various challenges that may arise [72]. Some areas of difficulty include: (a) inclusion of wrong areas or cells; (b) technical errors; and (c) heterogeneity in TIL distribution, etc. In addition to AI‐based methods, the feasibility of a radiomics model and the role of radiology have also been explored and could potentially offer benefits [73, 74].

Of interest are also studies that explore prediction scores incorporating TILs. TILs show potential for a better performance in predicting pCR [75]. It has been postulated that the peripheral blood neutrophil‐to‐lymphocyte ratio (NLR) may help to screen the high‐risk population of TNBC patients after neoadjuvant therapy [76]. On the one hand, low NLR was a strong indicator of outcome and was postulated to be useful for prognostication and disease monitoring [77]. On the other hand, Garcia‐Torralba et al. questioned the utility of NLR as a prognostic biomarker in early BC and found a lack of correlation of NLR with TME immune response [78].

The presence or absence of various immune cell subtypes can have various implications on the antitumor immune response. Cellularly, the proportion of CD4^+^ cells was reported to be higher in relapse than in primary tumors [79]. The PAMELA trial showed that multiple B‐cell‐related signatures were more strongly associated with pCR and event‐free survival (EFS) than TILs, which largely represent T‐cells [80]. Chen et al. found that markers such as CD47^+^ and CD68^+^ were associated with concurrent blood vessel invasion (CD31^+^) and interval‐presenting BC. Interestingly, they found that high combined expression of CD47‐CD68 was an independent prognostic factor associated with poor prognosis [81]. In a large observational study with 1054 BC patients, higher CD8^+^ T‐cells as well as TILs in the TME were associated with an improved long‐term survival outcome [82].

Practice guidelines have been introduced to improve interobserver agreement in TIL assessment among pathologists [2, 83]. Li et al. combined the tumor‐stroma‐ratio and sTILs to provide predictive information on pCR in NAC settings [84]. Their results showed that patients with low stromal area had more frequently pCR than those with high stromal area. This variable was also an independent predictor for pCR to NAC [84]. The study from Kang et al. showed that the TIL score and histological grade correlate. This means that the TIL score could potentially guide the therapeutic management of BC patients [85]. A recent paper investigated the predictive nature of TILs in correlation with PD‐L1 positivity. The authors identified TIL cut‐offs predictive of PD‐L1 positivity (the SP142 clone seemed to exhibit higher positivity rates compared to 22C3) [86].

The clinical utility of TILs scoring depends largely on how they are measured [1]. H&E‐based assessment remains the most widely used approach due to its accessibility and simplicity, but it lacks the ability to distinguish between immune cell subtypes [2]. Immunohistochemistry (IHC) allows for more specific identification of TIL subsets, such as cytotoxic or regulatory T‐cells, but it provides only semi‐quantitative data and limited spatial context [87]. In contrast, RNA sequencing—both bulk and single‐cell—offers a high‐resolution view of immune cell diversity and activation states, although these methods do not retain spatial information and can miss rare cell populations [88]. More recently, ST has emerged as a powerful tool that preserves tissue architecture while providing transcriptomic data, but it is costly and technically demanding [89]. Each of these methods brings distinct advantages and limitations, and understanding these trade‐offs is essential for interpreting study findings and advancing the clinical application of TIL profiling in BC.

### 2.6 Future Perspectives

Results from *KEYNOTE‐522* (K522) for patients with locally advanced TNBC showed that TILs are significantly associated with the pCR rate in the trial regimen (chemotherapy and pembrolizumab administered in the neoadjuvant setting) and may potentially serve as a biomarker to select optimal treatment [90].

Not only is the presence of TILs important, but their composition and distribution play a role as well. The importance of the spatial distribution of specific immune subsets was investigated, specifically examining CD103^+^ (a cell adhesion molecule important for T‐cells to be retained in the epithelial area) and FOXP3^+^ (a marker for regulatory T‐cells) [91]. CD103^+^ TILs were closer to tumor nests than FOXP3^+^ TILs in the tumor‐stromal interface. The densities of these cells were associated with high‐grade disease, TNBC, and stromal TILs. Similarly, Gonzales et al. stressed the importance of considering different immune cell types for classification in TNBC. The authors propose a new classification of TNBC immune infiltration with CD8^+^ T‐cell and plasma cell densities in the tumor center and infiltrative margin [92].

## 3 TILs in Endometrial Cancer

**FIGURE 3:**
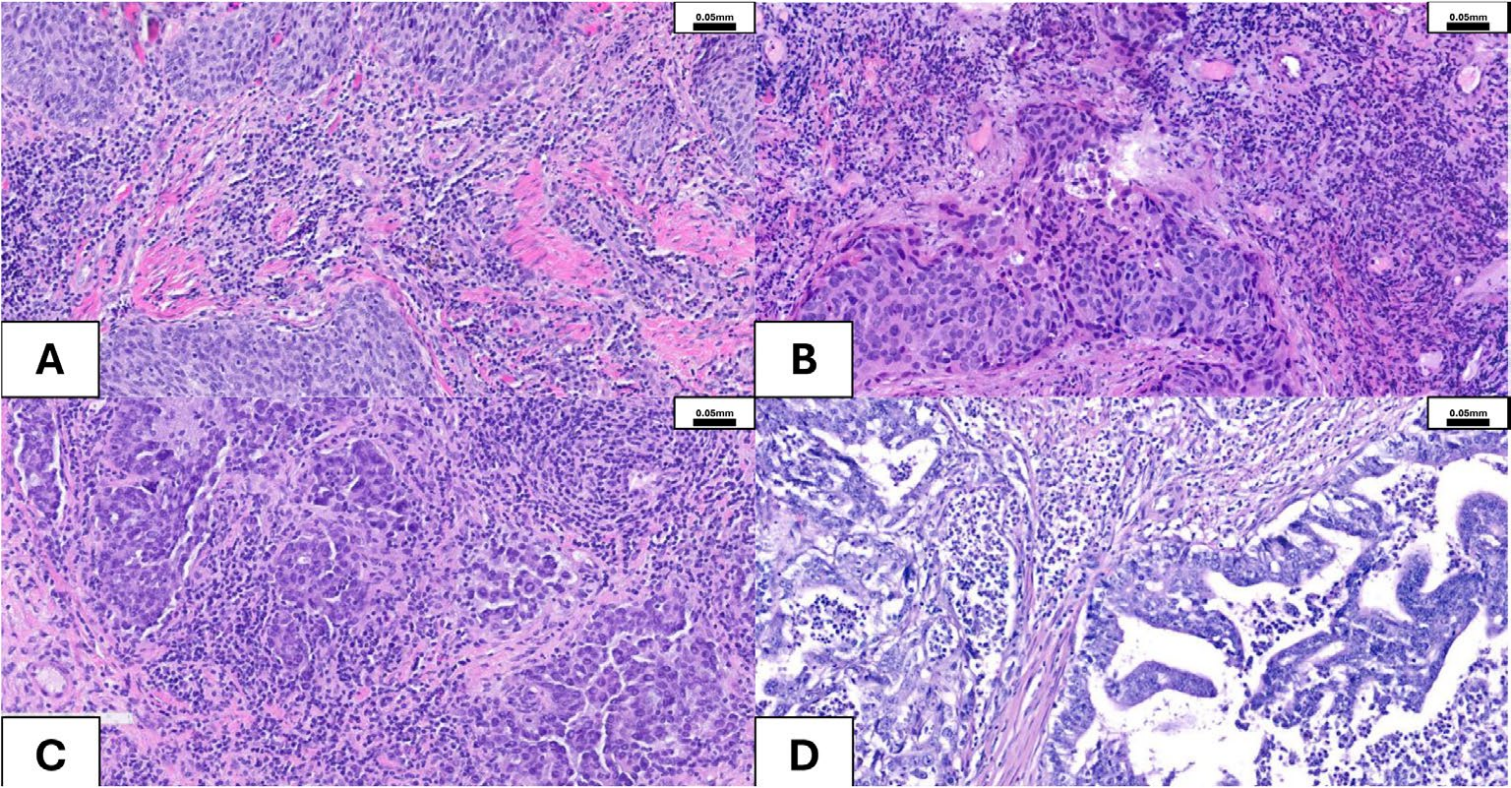
Vaginal, vulvar, ovarian, and endometrial cancer. (A) Squamous cell carcinoma (SCC) of the vulva, showing numerous TILs. (B) HPV‐dependent squamous cell carcinoma (SCC) of the vagina, showing numerous TILs. (C) High‐grade serous carcinoma (HGSC) with surrounding inflammatory cells. (D) Poorly differentiated endometrioid adenocarcinoma of the uterus (Grade 3), with a pathogenic mutation of the POLE gene, showing numerous tumor‐infiltrating lymphocytes (TILs).

Similar to TILs in BC described above, TIL content has different prognostic and predictive associations in different subtypes of endometrial carcinoma (EC), which include *POLE*‐mutated (POLEmut), mismatch‐repair deficient (MMRd), p53‐mutated/abnormal (p53abn), and no specific molecular profile (NSMP) [93, 94, 95]. Much of the research published to date on EC was prior to the full adoption of molecular stratification in EC, thereby limiting the utility of TIL analysis for prognosis within subtypes. For example, POLEmut ECs (Figure 3A) have mutations in the proofreading portion of the DNA‐polymerase enzyme, leading to thousands of mutations, abundant potential immunological mutations, and an excellent prognosis [96, 97] (Table 1). These tumors also have high TIL counts compared to other subtypes [93, 97]; thus, TIL studies that do not stratify EC into molecular subtypes suffer from the confounding effect that TILs are prevalent in the best prognostic group. Within the POLEmut subtype, outcomes are so favorable, regardless of immune infiltration, that TIL density is unlikely to impact survival [98]. MMRd ECs have errors in DNA repair enzymes and generate hundreds of mutations. MMRd ECs respond extremely well to immune checkpoint inhibitors in multiple clinical trials [99, 100, 101], and ICIs are FDA approved for this EC subtype [102]. Within the MMRd subtype, increased CD8+ TILs have been found to associate with response to therapy in some studies [103], but responders were also present in TIL low cases, meaning there is limited utility in using TIL density for treatment decisions [104]. Interestingly, a recent ICI clinical trial found that some p53abn ECs also respond to immunotherapy, even though the mutation burden is an order of magnitude less than POLE‐mutated and MMRd subtypes [105]. Our research has demonstrated that increased TIL is associated with improved prognosis in p53abn EC [106], particularly in high‐stage disease; however, additional subgroup analysis is required to determine whether TIL density predicts response to ICI in p53abn EC. Finally, NSMP failed to respond to ICI [107], yet TILs in the invasive margin of this subtype have been found to be prognostic of overall survival (OS) [108]. Overall, the prognostic effect of TIL must be evaluated within specific subtypes of EC to be used clinically to guide therapy, and research into TIL within molecular subtypes is currently relatively immature.

The single‐cell landscape of endometrial cancer has also been investigated [109]: The endometrium and its various cell types undergo various forms of differentiation and remodel at a rapid rate during each menstrual cycle. If this homeostasis is disrupted, lesions occur. Ren et al. demonstrated that EC originates from the unciliated glandular epithelial cells [109]. Prognostically favorable cytotoxic CD8^+^ T‐cells and NK cells were prominent in normal endometrium [110], whereas CD4^+^ regulatory T cells (Treg), exhausted CD4,^+^ and exhausted CD8^+^ T‐cells dominated the tumors. CXCL3^+^ macrophages associated with M2‐polarization and angiogenesis were exclusively found in tumors.

## 4 TILs in Ovarian Cancer

The first paper to describe the importance of TILs in ovarian carcinoma was published more than 20 years ago [111]. Subsequent publications provided supporting evidence and refined the clinicopathologic features linked to strong TIL responses (e.g., CD3^+^ TILs [112]). Single‐cell transcriptomics could reveal a multitude of T‐cell subsets [113] with distinct cell states linked to immune infiltration [114] in ovarian cancer. In the past two decades, the number of intraepithelial CD8^+^ TILs has been identified as an independent prognostic factor in epithelial ovarian cancer, although different cut‐offs have been reported [115, 116, 117]. More recently, Spagnol et al. reported a significant increase in TILs, intraepithelial TILs (ieTILs), and CD8^+^ subpopulations following NAC [118], though this is only occasionally linked to improvement in progression‐free survival (PFS) and OS [119]. Marachenko et al. demonstrated, using IHC on tissue microarrays with subsequent digital/computational quantification, that higher levels of T‐regulatory cells (T‐regs) in high‐grade serous carcinoma (HGSC; Figure 3B), both in intratumoral and stromal areas, are associated with significantly increased OS [120]. While increased T‐reg infiltration is usually considered a poor prognosticator due to the well‐described immunosuppressive effects of this cell type, the literature in ovarian cancer seems conflicting: some studies support the association of higher T‐reg cell infiltration with improved outcome [121, 122], while others claim the opposite [123, 124, 125]. Not only their location, but also the phenotype of T‐regs (“effector” phenotype [126]) and microenvironmental stimuli (e.g., cytokines) [123] seem to (re)program their biological effects in a complex TME. ST could confirm compartmentalization of the TME [127, 128] with distinct differences for different tumor subclones [129] in ovarian cancer.

In ovarian clear cell carcinoma (OCCC), CD8^+^ T‐cell infiltration is associated with improved survival and inversely correlated with hypoxia [130], whereas high TILs (≥ 50/mm^2^) and PD‐L1 positivity (Combined Positive Score) predict shorter disease‐specific survival [131]. Fu et al. confirmed the prognostic value of CD8^+^ cytotoxic T‐cells in OCCC and described higher CD8^+^ T‐cell abundance in adjacent nonneoplastic tissue [132]. Microsatellite‐unstable OCCC revealed higher CD8^+^ T‐cell infiltration and consistent PD‐L1 expression [133]. However, as a potential mechanism of immune evasion, spatial profiling found evidence for immune mimicry (cancer cells adopting traits of immune cells) in OCCC [134]. Notably (high‐stage), mucinous ovarian cancer seems to be rather “immune‐cold” [125] but can still demonstrate PD‐L1 protein expression [135]. In endometrioid ovarian carcinoma, the presence of intraepithelial CD8^+^ T‐cells was described to be lower than in serous carcinoma and not associated with improved disease‐specific survival [136]. In contrast, Gallego et al. observed that, after adjusting for age, patients with localized endometrioid ovarian carcinomas who had moderate to high levels of intraepithelial CD8^+^ TILs experienced longer overall survival [137]. Malignant ovarian tumors exhibit significantly higher TIL infiltration than borderline tumors, and CD3^+^ and CD20^+^ TIL counts negatively correlate with survival [138], while high CD3^+^CD137^+^ TIL frequencies (> 9.6%) were linked to longer OS, independent of germline mutation or debulking status [139]. Surprisingly, to the best of our knowledge, no comprehensive studies on TILs in primary peritoneal and fallopian tube cancer have been published. Nevertheless, single‐cell profiling could reveal decreasing immune cell interaction in the fallopian tube after menopause [140].

Finally, recent work assessed the efficacy and safety of autologous TILs in recurrent or refractory ovarian cancer, colorectal cancer (CRC), and pancreatic ductal adenocarcinoma. They concluded that TIL manufacturing with the assistance of 4‐1BB (CD137) and CD3 agonism is feasible. While no responses were observed, a significant portion of patients achieved stable disease, suggesting early/partial immunological effect [141].

## 5 TILs in Cervical Cancer

Cervical cancer (Figure 3C) incidence has dropped by more than half since the 1970s due to widespread screening and treatment, though overall rates have stabilized recently [142]. In women aged 30 to 44 years, however, incidence rates have increased by 1.7% annually from 2012 to 2019 [142].

Advances in research on TILs in cervical cancer are promising, though data are more limited compared to other cancers like melanoma and BC. Studies focus on the prognostic value of TILs, including their types, numbers, and functionality. Recent efforts in TIL‐based therapy target HPV‐specific TILs and explore ways to enhance their immune functions.

He et al. [143] tried to predict the prognosis of cervical cancer by constructing an immunoscore model with five specific immune cell types. They saw that neutrophils, activated mast cells, and macrophages were worse prognostic indicators, while CD8^+^ T‐lymphocytes were favorable. They concluded that their immunoscore might be an effective predictive tool to distinguish patients who might benefit from immunotherapy [143]. Ohno et al. showed that infiltration with CD8^+^ TILs was associated with the presence of pelvic lymph node metastasis. Abundant infiltration by CD3^+^, CD4^+^, CD8^+^, CD206^+^, and FOXP3^+^ TILs was a statistically significant indicator of better PFS and OS [144]. Furthermore, in another study, the authors showed that TIL mono‐therapy can be a promising treatment strategy for patients with late‐stage metastatic cervical cancer, even with severe myelosuppression [145]. Wild et al. set out to quantify tumor‐infiltrating immune cells in a panel of 238 sporadic cervical cancers and investigate the correlation between cervical cancer subtype and patient survival. The amount of TILs was significantly increased in cervical SCC (Figure 3B) in comparison to cervical adenocarcinoma. In cervical SCC, TILs infiltration showed a negative correlation with age, FIGO stage, and the hypermethylation of the histone protein H3 (H3K4me3). Additionally, immune infiltration was an independent positive prognosticator for DFS in patients with cervical SCC. Patients with the strongest TIL infiltration showed better DFS [146].

Li et al. performed single‐cell transcriptomic analysis on three cervical SCC cases and proposed a molecular stratification [147]. They reported extensive heterogeneity of the malignant cells and, after clustering, epithelial subpopulations exhibited different genomic and transcriptomic signatures. They also identified CAFs, inflammatory (iCAF), and myofibroblastic (myCAF), which could promote tumor progression. Furthermore, they found two subpopulations of CD8^+^ T‐cells (proliferative—MKI67^+^ and non‐cycling exhausted PDCD1^+^). Putting these data together, they define four different molecular subtypes, namely hypoxia, proliferation, differentiation, and the immunoactive subtype. The hypoxia subtype showed the worst prognosis, while patients of the immunoactive subtype had the longest overall survival [147]. Sheng et al. established a single‐cell transcriptional atlas on cervical exfoliated cells and performed scRNA‐seq of 56,173 cervical exfoliated cells from 15 samples (e.g., normal cervix, low‐grade squamous intraepithelial lesion (LSIL), high‐grade squamous intraepithelial lesion (HSIL), and tumor cells of different differentiations). They showed that cells in LSIL were composed of a large part of cytotoxic T‐cells and NK cells. Additionally, the LSIL group presented a higher CD8 cytotoxic score compared with HSIL and the invasive carcinoma groups [148].

## 6 TILs in Vulvar Cancer

TILs have gained comparatively little attention in vulvar cancer (Figure 3D; compare Table S1). Broadly speaking, the malignancy of the vulva is mainly SCC (and its precursor lesions) and malignant melanoma, with other lineages being exceedingly rare. ICI has shown promise in vulvar SCC. Previous research suggests combining PD‐1, PD‐L1, and TIM‐3 inhibitors for potentially better outcomes [149]. PD‐L1 is commonly expressed in vulvar SCC (vSCC) [150], regardless of p16‐status. Elevated levels of PD‐L1 expression in vSCC were linked to poor prognosis, while p16 positivity emerged as a separate factor associated with improved outcomes [151]. High‐risk HPV status has been shown not to correlate with immune infiltration [152]. The first ST study in vulvar SCC [153] found significantly more immune cells in HPV‐independent SCC than in HPV‐dependent SCC. The authors found a higher abundance of CD45^+^ immune cells, particularly CD4^+^ resting memory and follicular helper T‐cells within SCC regions. Immune cell phenotypes shifted from resting states in pre‐invasive lesions to activated states in SCC and surrounding inflammatory zones [153].

Interestingly, tumoral PD‐L1 expression is rather low in vulvar melanoma, but if expressed, it correlates well with the density of peritumoral CD8^+^ and FOXP3^+^ lymphocytes [154]. In vulvar Paget's disease, the immune infiltrate seems more pronounced in the stroma than in the epithelium [155]. In early‐stage vulvar cancer, increased activated (CD3^+^ PD‐1^+^) helper T‐cell counts were correlated with improved prognosis, regardless of HPV‐ or p53‐status [156]. Active immune signaling is present in about a third of vSCC, suggesting potential for neoadjuvant PD‐1/PD‐L1 immunotherapy [157]. Pembrolizumab showed some benefit in PD‐1^+^ vaginal and vulvar SCC [158]. Lastly, low CD8^+^ TIL counts were shown to be an adverse prognostic factor for genital melanomas [159].

## 7 Discussion and Perspective

Higher levels of TIL infiltration (immune “hot”) are frequently associated with a better prognosis, suggesting a stronger anti‐tumor immune response. Accordingly, low TIL levels or compromised immune infiltration can be signs of a worse(ning) prognosis. In BC, higher TIL counts are generally linked to improved survival, but, for instance, in DCIS increased TIL counts have been associated with a poorer prognosis. TIL counts and their prognostic power rely on a multitude of factors, though, e.g., immune cell subtype combinations and the interplay with other TME elements, molecular profile, spatial density, and even ethnicity (as evidenced above).

We focused on TILs in general as a marker for susceptibility to immunotherapy because IHC for CD3, CD8, CD4, and CD20 is readily available in the pathology lab. However, mechanistically, the role of an individual T‐cell within TILs may be affected by its expression of cell exhaustion molecules like PD‐1, CTLA‐4, LAG3, TIGIT, CD39, and TIM3 [160, 161]. Although binding to cognate ligands leads to deactivation of the T‐cell, these proteins are also markers of T‐cells capable of recognizing the tumor cells and are exhausted due to chronic stimulation [160]. The nuances of exhaustion markers and their utility as markers for immunotherapy susceptibility are relatively early in investigations and are reviewed here [160], with limited studies of their relevance in lower gynecological and BC [162, 163].

In endometrial cancer, particularly the *POLE*‐ultramutated subgroup, there is a clear correlation between higher TIL counts and improved survival. The same holds true for invasive ovarian epithelial tumors with higher CD8^+^ T‐cell counts. Autologous TIL transfer shows promise as personalized immunotherapy, as tested, for instance, in recurrent, refractory ovarian cancer. In cervical cancer, attempts have been made to combine different immune cell types to better stratify patient prognosis. Research on TILs in rarer tumors such as vaginal, vulvar, and fallopian tube cancers remains limited. These malignancies, though underexplored, may benefit from checkpoint inhibitors as shown for vulvar and vaginal SCC in smaller cohorts (see above and in Table S1).

TIL therapy has shown promising results despite being costly and complex. Apart from melanoma, TIL therapy has been proven to have strong clinical benefits for patients with cervical cancer [164, 165, 166] and has also shown preliminary efficacy in CRC [167], cholangiocarcinoma [168], non‐small cell lung cancer [169], and BC [170]. Despite ovarian cancer, its potential for gynecological cancers has not been explored.

To conclude, in‐depth research to deconvolute the TME in gynecologic malignancies is needed. The apparent but rather anecdotally reported benefit of immunotherapy in these deadly cancers is promising but needs urgent translation to trials.

## Author Contributions

All authors (K.S., U.M., S.D.M., and K.B.) drafted, revised, and substantially contributed to the conception of the manuscript and approved its submission. K.S. and U.M. are joint first authors.

## Ethics Statement

The authors have nothing to report.

## Conflicts of Interest

The authors declare no conflicts of interest.

## Supporting information


**Supplementary Table 1.** Overview of key studies on TILs in breast and female genital tract cancers.

## Data Availability

The authors have nothing to report.
