# Supplementary material for: Tumor‐Infiltrating Lymphocytes in Breast and Female Genital Tract Cancers: Overlooked Potential and Unexplored Frontiers
Source: Cancer Med. 2025 Jul 7;14(13):e71023. doi: 10.1002/cam4.71023 (PMC12230770; doi:10.1002/cam4.71023)
Supplement: Supplementary file 1 — Supplementary Table 1. Overview of key studies on TILs in breast and female genital tract cancers. [file CAM4-14-e71023-s001.docx]

**Supplementary Table 1:** Overview of key studies on TILs in breast and female genital tract cancers.

| **Cancer type** | **Study** | **Number of cases examined** | **Tumor histology/subtype** | **Disease stage** | **TILs quantification method** | **TILs subtype** | **Clinical correlation and outcome** | **Comments/limitations** |
| --- | --- | --- | --- | --- | --- | --- | --- | --- |
| **Breast cancer** | Denkert et al., 2018 | 3771 | Luminal A/B, Her2-enriched and triple-negative | Stage I, II and III, no distant metastasis | Manual quantification on core biopsy based on recommendation of TIL working group | TILs in general, no particular subtype analyzed | Increased TIL concentration predicted response to neoadjuvant chemotherapy in all molecular subtypes; survival benefit in HER2-positive breast cancer and TNBC; adverse prognostic factor for survival in luminal-HER2-negative breast cancer | Prospective study, large cohort |
|  | Stanton et al. 2016 | 13914 | Luminal A/B, Her2-enriched and triple-negative | Not specified | Different methods depending on the studies included (mainly manual quantification) | TILs in general, CD8 and FOXP3 | Magnitude of TIL is variable within and between breast cancer subtypes (highest in triple-negative) | Systematic review |
|  | Denkert et al. 2010 | 1058 | Triple-negative | Nodal positive and nodal negative, no distant metastasis | Manual quantification | CD3/CD20 | Presence of TILs predict response to neoadjuvant chemotherapy | Prospective study, large cohort |
|  | Leon-Ferre et al., 2024 | 1966 | Triple-negative | Nodal positive and nodal negative, no distant metastasis | Manual quantification on histological slide based on recommendation of TILs working group | TILs in general, no particular subtype analyzed | Early-stage TNBC who did not undergo adjuvant or neoadjuvant chemotherapy, breast cancer tissue with a higher abundance of TIL levels was associated with significantly better survival | Retrospective multicentric study |
|  | De Jong et al., 2022 | 441 | Triple-negative | Nodal negative | Manual quantification according to pre-established cut-offs | TILs in general, no particular subtype analyzed | Chemotherapy-naïve, young patients with N0 TNBC with high sTILs (≥ 75%) have an excellent long-term prognosis | Retrospective study, focused on a particular subgroup of triple-negative breast cancer |
|  | Ray et al., 2022 | 80 | Triple-negative | Nodal positive and negative treated with neoadjuvant chemotherapy | Manual quantification on core biopsies | CD3/CD4/CD8/CD20 | TIL profile with low CD3, CD4, CD20, and CD56 expression predicts poor pathological response to neoadjuvant chemotherapy | Prospective study, small cohort |
|  | Vaid et al., 2022 | 229 | Luminal A/B, Her2-enriched and triple-negative | Nodal positive and negative patient | Manual quantification on core biopsies | TILs in general | Higher TILs in triple-negative and Her2-enriched; better outcomes in tumors with higher TILs | Retrospective study, heterogeneous cohort with all molecular subtypes included |
|  | Li et al., 2022 | 7866 (29 studies) | Luminal A/B, Her2-enriched and triple-negative | Not specified | Many methods depending on the study (mostly manual quantification) | TILs in general | Increased levels of TILs are associated with increased rates of response to neoadjuvant chemotherapy and improved prognosis for the molecular subtypes of triple-negative and HER2-positive breast cancer, but not for patients with hormone receptor positive breast cancer. A threshold of 20% TILs was the most powerful outcome prognosticator of pathological complete response. | Meta-Analysis |
|  | Qian et al., 2023 | 991 | Luminal A/B, Her2-enriched and triple-negative | Not specified | Manual quantification on histology | TILS in general | Higher TILs associate with better response to chemotherapy in triple negative and Her2-positive | Retrospective study, focus on response to neoadjuvant chemotherapy |
|  | Jääskeläinen et al., 2023 | 139 | Her2-enriched | Stage I, II and III, no distant metastasis | Quantification with QuPath software on digital slides | FoxP3/CD8 | Higher FoxP3 associated with shorted disease-free survival; higher CD8+ associate with response to trastuzumab | Retrospective study |
|  | Lu et al., 2023 | 973 | Her2-0 and Her2-low | Nodal positive and negative | Manual assessment on histology | TILs in general | TILs increment was independently associated with favorable survival in the HER2-low group | Retrospective study, focus mainly on differences between Her2-0 ad Her2-low, not n TILs, heterogeneous and large cohort |
|  | Baez-Navarro et al., 2024 | 977 | Her2-low and Her2-0 | Nodal positive and negative; some cases with unknown staging | Manual assessment on histology | TILs in general | No significant difference in TILs density or complete pathologic response rate was found between HER2-0 and HER2-low cases | Retrospective multicentric study |
|  | Fukui et al., 2023 | 170 | Hormone receptor positive, Her2-negative | Nodal positive and negative, no distant metastasis | Manual assessment on core biopsy | CD8/FoxP3 | Post-treatment TILs associate with response to neoadjuvant endocrine therapy | Retrospective study |
|  | Albusayli et al., 2023 | 429 | Triple-negative | Stage I, II, III | Computational pathology on digital slides | TILs in general | Automatic quantification of TILs robust predictive and prognostic biomarker in TNBC | Retrospective study, computational quantification |
|  | Sayed et al., 2023 | 226 | Luminal A/B, Her2-enriched and triple negative included; histological subtypes not specified | Stage I, II, III and IV | Semi-quantitative quantification on tissue microarray | CD3/CD4/CD8/CD20 | TILs associated with high tumor grade and aggressive behavior | Retrospective study, focus on one ethnic group, quantification on tissue microarray |
|  | Dimitrakopoulos et al., 2024 | 1024 | Different histological subtypes (NST, lobular, mucinous); Luminal A/B, Her2-enriched and triple negative included | Nodal negative and nodal positive cases, no distant metastasis | Manual quantification on histology | CD8 | Higher CD8+ T cells as well as TILs in the tumor microenvironment associated with an improved long-term survival outcome | Prospective study, focus on effects of chemotherapy |
|  | Kang et al., 2024 | 556 | Histological subtype not specified; Luminal A/B, Her2-enriched and triple-negative included | Nodal negative and nodal positive cases, no distant metastasis | Multiphoton imaging system and data acquisition | TILs in general | TILs-score correlated with histological grade | Retrospective study, large cohort, focus on prediction of tumor grading based on TILs |
|  | Suwannaphoom et al., 2024 | 141 | NST (triple negative) | Not specified | Manual quantification on histology in adherence to the 2014 guidelines set by the International TILs Working Group | TILs in general | TILs cut-offs predictive of PD-L1 positivity | Retrospective study, focused on correlation between TILs and PD-L1 but not with clinical data |
|  | Wood et al., 2024 | 76 | NST | Nodal negative or positive, no distant metastasis | Manual quantification on histology | TILs in general | High TILs associate with complete pathological response | Retrospective study, focus on triple-negative breast cancer treated according to KEYNOTE-522 |
|  | Chan et al., 2024 | 265 | Many histological subtypes (NST, lobular, metaplastic and mucinous | Stage I, II and III | Digital quantification | CD103/FoxP3 | High sFoxP3 TIL density and the lower distance of CD103 TILs from the tumor nests had independent favorable prognostic values | Retrospective study, evaluates the spatial distribution of TILs, heterogeneous cohort for histology and staging |
| **Endometrial cancer** | Palomero et al., 2022 | 47 | Endometrioid carcinoma | Different stages (I, II, III and IV) | Flow cytometry, single-sample Gene Set Enrichment Analysis (ssGSEA) based on the expression of 28 immune cell marker genes | CD3/CD39/TIM-3/CXCL-13/PD-1 | Endometrial cancer frequently infiltrated by tumor-reactive TILs, expression of PD-1hi and CD39 or PD-1hi can be used to select and expand CD8+ and CD4+ tumor-reactive TILs, respectively | Transcriptomic-based data, retrospective study |
|  | Biase et al., 2025 | 213 | Endometrioid carcinoma (POLE-mutated, microsatellite instable, p53-mutated, non-special type) | Different stages (I, II, III and IV) | quantified by digital image analysis | CD3/CD20/CD8 | Immune infiltrate more frequent in mismatch-repair deficient tumors and POLE mutated, in non-special type tumors TILs can predict survival | Retrospective study, stratification per molecular subtypes |
| **Ovarian cancer** | Zhang et al., 2003 | 186 | Serous, mucinous and clear cell carcinoma | Stage III and IV | Manual quantification on histology | CD3/CD4/CD8/CD19/CD57 | Presence of CD3+ TILs associated with progression-free and overall-survival | Retrospective study, focus on advanced cancer |
|  | Morse et al., 2019 | 250 | High-grade serous carcinoma | Different stages (I, II, III and IV) | Manual quantification on histology | CD3 | TILs more frequent in homologous-recombination deficient tumors; higher TILs associated with improved overall survival | Retrospective study |
|  | Goode et al., 2017 | 5500 | Different subtypes (high-grade serous, endometrioid, mucinous and clear cell) | Different stages (I, II, III and IV) | Manual quantification on histology | CD8 | CD8+ TILs associated with longer overall survival in serous high-grade carcinoma | Multicentric observational study, large cohort |
|  | Hwang et al., 2012 | 1815 | Different histological subtypes, mostly serous carcinoma | Stage III and IV | All studies included scored intraepithelial TIL based on immunohistochemical analysis of tumor specimens: two used cryosections, five used paraffin-embedded tissue, and three used tissue microarrays (TMA) | CD3/CD8 | Lack of intraepithelial TILs is significantly associated with a worse survival among patients | Meta-Analysis |
|  | Marchenko et al., 2023 | 222 | High-grade serous carcinoma | Different stages (I, II, III and IV) | Digital assessment with open-source Software QuPath (v0.2.0-m10) on tissue microarray | CD25 FoxP3 (Tregs) | Higher Tregs were associated with a better overall survival | Retrospective study, quantification on tissue microarray, heterogeneous cohort for staging |
|  | Milne et al., 2009 | 487 (199 serous carcinoma; 288 of other subtype) | Different subtypes (high-grade serous, endometrioid, mucinous and clear cell) | Different stages (I, II and III), no metastatic stage included | Manual quantification on tissue microarray | CD3/CD4/CD8/CD25/TIA-1/Granzyme/FoxP3/CD20 | In high-grade serous carcinoma disease-specific survival associated with the markers CD8, CD3, FoxP3, TIA-1, CD20 | Retrospective study, quantification on tissue microarray, heterogeneous cohort for histology and staging |
|  | Leffers et al., 2009 | 306 | Different subtypes (serous, mucinous, endometrioid, clear cell carcinoma) | Different stages (I, II, III and IV) | Manual quantification on tissue microarray | CD8/FOXP3 | T-lymphocytes infiltrating primary and metastatic ovarian cancer sites are associated with improved prognosis | Retrospective study, quantification on tissue microarray, heterogeneous cohort for histology and staging |
|  | Meagher et al., 2023 | 126 | Mucinous carcinoma | Different stages (I, II, III and IV) | Manual quantification on tissue microarray | CD3/CD8/CD20/CD79A/FOXP3 | High epithelial density of CD8+/FOXP3+ associated with poorer survival; high epithelial CD79a + plasma cells conferred better survival | Retrospective study, quantification on tissue microarray, univariate analysis only |
|  | Guo et al., 2023 | 218 | Clear cell carcinoma | Different stages (I, II, III and IV) | Manual quantification on tissue microarray | CD8 | CD8 + T cell infiltration positively predicted survival and negatively correlates with hypoxia | Retrospective study, quantification on tissue microarray |
|  | Lin et al., 2023 | 76 | Clear cell carcinoma | Different stages (I, II, III and IV) | Manual quantification on tissue microarray | CD8 | Higher CD8 + TILs associated with shorter disease-specific and overall survival. PD-L1 positivity associated with fewer CD8+ TILs | Retrospective study, quantification on tissue microarray |
|  | Fu et al., 2025 | 18 | Clear cell carcinoma | Different stages (I, II, III, IV) | Quantitative pathological analysis software | CD8 | Difference in cell density between CD8+ non-tumor-infiltrating lymphocytes and CD8+ (TILs) exceeding 70 cells/mm2 associated with poorer progression-free survival | Retrospective study, small cohort |
|  | Howitt et al., 2017 | 30 | Clear cell carcinoma | Not reported | Manual assessment on histology | CD3/CD4/CD8 | Microsatellite instability associated with higher CD8+ TILs | Retrospective study, small cohort, no correlation with clinical data |
|  | Clark et al., 2009 | 500 (retrospective)  40 (prospective) | Serous, endometrioid, clear cell, mucinous | Different stages (I, II and III), no metastatic stage included | Manual assessment on tissue microarray (dicotomic distinction between present or absent) | CD3/CD4/CD8 | Retrospective cohort: presence of intraepithelial CD8(+) T-cells correlated with improved disease-specific survival. Prospective cohort: intraepithelial CD8(+) T-cells correlated with the presence of mutation or loss of expression of BRCA1 through promoter methylation | Quantification on microarray, dichotomic distinction between present or absent |
|  | Gallego et al., 2022 | 119 | Endometrioid and clear cell carcinoma | Different stages (I, II, III and IV) | Manual assessment on tissue microarray | CD3/CD8 | Intraepithelial CD8 associated with improved outcome in endometrioid and clear cell carcinoma | Retrospective study, quantification on microarray |
|  | Stout et al., 2024 | 138 | Different subtypes (clear cell, endometrioid, mucinous, serous) | Different stages (I, II, III and IV) | Manual visual assessment | CD3/CD20 | Stromal CD3 and CD20 correlated negatively with survival. Malignant ovarian tumours significantly higher infiltration of immune cells than borderline tumours | Retrospective study, heterogeneous cohort for histology and staging |
|  | Tubridy et al., 2024 | 47 | Serous carcinoma | Most cases (85%) Stage III or IV | Flow cytometry on viably banked ovarian cancer digests | CD3/CD8/CD137 | Prevalence of CD3+ CD137+ TILs in digested OC specimens is associated with improved OS, while general TIL markers are not | Retrospective study, small cohort, TILs quantified only with cytometry (no information on spatial distribution) |
| **Cervical cancer** | Ohno et al., 2020 | 55 | Squamous cell carcinoma | Locally advanced | Automatic scoring with computerized image analysis system | CD3/CD4/CD8/CD20/FoxP3/CD206 | Infiltration with CD8+ TILs associated with pelvic lymph node metastasis. Infiltration by CD3+, CD4+, CD8+, CD206+, and FOXP3+ TILs indicators of better progression-free and overall survival | Retrospective study, small cohort |
|  | Wild et al., 2023 | 238 | Squamous cell carcinoma and adenocarcinoma | Locally advanced, no distant metastasis | Manual semi-quantitative quantification on histology | Generic TILs (no further distinction) | independent positive prognosticator for disease-free survival (DFS) in patients with squamous cell carcinoma | Retrospective study, relatively large cohort, no distinction between TILs subtype, semi-quantitative evaluation |
| **Vulvar cancer** | Cocks et al., 2020 | 21 | Squamous cell carcinoma | Locally advanced | Manual quantification on histology | PD-1/CD8/FoxP3 | higher disease recurrence and cancer mortality in tumors with higher CD8 expression | Retrospective study, small cohort, heterogeneous cohort (different stages, different ethnicities) |
|  | Linden et al., 2018 | 10 | Paget disease | In situ | Manual quantification on digital slides | CD8/FoxP3/CD56 | Decreased immune cells in epithelium and an abundant number of immune cells in the stroma, consisting predominantly of T-cells | Small cohort, retrospective study, considers only in situ disease, evaluated only changes in immune environment after therapy but not the response to therapy or prognosis |
|  | Kortekaas et al., 2019 | 65 | Squamous cell carcinoma | Locally advanced, no distant metastasis | Computational pathology on digital slides | CD3/CD8/FOXP3 | p53 + vulvar carcinoma mostly are cold tumors whereas HPV+ are strongly T-cell infiltrated; High numbers of intraepithelial CD3+ and CD3+CD8-FOXP3- T cells are associated with longer overall survival and recurrence-free period | Retrospective studies, reproducible |
|  | Chlopik et al., 2018 | 75 | Melanoma | Locally advanced and metastatic | Dicotomic score (low vs. high) on histology | CD8/FoxP3 | high peritumoral FOXP3+ lymphocytes independently predicted better melanoma-specific survival | Retrospective study, Multicentric, follow-up data available, heterogeneous cohort |
|  | Yu et al., 2020 | 55 | Melanoma | Locally advanced | Manual scoring on histology | CD8 | Low CD8+ tumor-infiltrating lymphocyte density were adverse prognostic factors | Small cohort, detailed information on staging missing, retrospective study |
